# The influence of caffeinated and non-caffeinated multi-ingredient pre-workout supplements on resistance exercise performance and subjective outcomes

**DOI:** 10.1080/15502783.2022.2060048

**Published:** 2022-04-04

**Authors:** Matthew T. Stratton, Madelin R. Siedler, Patrick S. Harty, Christian Rodriguez, Jake R. Boykin, Jacob J. Green, Dale S. Keith, Sarah J. White, Brielle DeHaven, Abegale D. Williams, Grant M. Tinsley

**Affiliations:** Energy Balance & Body Composition Laboratory; Department of Kinesiology & Sport Management, Texas Tech University, Lubbock, TX, USA

**Keywords:** Caffeine, stimulant, non-stimulant, citrulline, betaine, beta-alanine, alpha-glyceryl phosphoryl choline

## Abstract

**Background:**

There is substantial consumer and practitioner interest in an emerging supplement class known as multi-ingredient pre-workout supplements (MIPS), largely due to their prevalence in resistance training communities as well as research findings demonstrating the ergogenic impact of caffeine on muscular performance. However, limited research has examined the potential efficacy of non-caffeinated MIPS, despite their growing popularity among those who are caffeine-sensitive or who train later in the day.

**Methods:**

Twenty-four resistance-trained college-aged males (n = 12) and females (n = 12) completed three visits in which they ingested either a caffeinated MIPS (C), an otherwise identical non-caffeinated MIPS (NC), or placebo in a double-blind, counterbalanced, crossover fashion. Squat isometric peak force (PF_iso_), rate of force development (RFD), and isokinetic performance were assessed. Upper and lower body maximal muscular strength and endurance were evaluated using the bench press and leg press, respectively. Visual analog scales for energy, focus, and fatigue were completed five times throughout the testing protocol. The effects of supplementation and biological sex on all variables were examined using linear mixed effects models.

**Results:**

Significantly greater PF_iso_ was observed in both C (*b*: 0.36 transformed units [0.09, 0.62]) and NC (*b*: 0.32 transformed units [95% CI: 0.05, 0.58]) conditions, relative to placebo. Early RFD (RFD_50_) may have been higher with supplementation, particularly in females, with no effects for late RFD (RFD_200_) or peak RFD. In addition, increases in subjective energy after supplement ingestion were noted for C, but not NC. No effects of supplementation on traditional resistance exercise performance or isokinetic squat performance were observed, other than a lower leg press one-repetition maximum for males in the NC condition.

**Conclusions:**

These data indicate that acute ingestion of either a caffeinated or non-caffeinated pre-workout formulation improved maximal force production during an isometric squat test but did not provide additional benefit to leg press, bench press, or isokinetic squat performance over placebo, within the context of a laboratory environment. The consumption of a caffeinated, but not non-caffeinated, MIPS increased subjective ratings of energy over placebo when assessed as part of a testing battery.

## 1. Introduction

The regular use of dietary supplements has become increasingly common. Currently, it is estimated that the global economic impact of the dietary supplement market will expand by approximately 8.6% per year, potentially reaching 272.4 Billion USD by the year 2028 [1]. A previous review of findings from the National Health and Nutrition Examination Survey (NHANES) by Kantor et al. [2] suggested that approximately 50% of US adults regularly consume one or more dietary supplements. Similarly, a 2020 survey by the Council for Responsible Nutrition found that 73% of respondents reported utilizing nutritional supplements in some manner, with 30% specifically reporting the use of ‘sports supplements’ [3]. These findings demonstrated a notable increase over the results from the same survey conducted in 2016, in which only 20% reported using ‘sports supplements’, though similar overall supplement usage was found [4].

Of particular interest in the field of sports supplements is the growing category of multi-ingredient pre-workout supplements (MIPS). Previously, a review by Harty and colleagues [5] suggested the ingestion of MIPS on an acute or longitudinal basis may have the ability to increase subjective ratings of energy as well as exercise performance outcomes such as muscular endurance. These effects, along with substantial marketing efforts by supplement manufacturers, may help explain the increased interest in MIPS. Indeed, a 2019 survey [6] of 872 regular MIPS users (defined as repeated use over the prior 3 months) found the three most common self-reported reasons for ingesting MIPS were increased energy, muscular endurance, and a better ‘pump’.

The reported exercise performance benefits noted with MIPS ingestion are often attributed to caffeine content. A 2019 review of the ingredient profiles of the top 100 selling MIPS on a commercial supplement website found that 86% of products contained caffeine, with an average dose of 254 mg per serving [7]. Investigations of other common ingredients in MIPS, such as citrulline [8,9], beta-alanine [10,11], and taurine [12,13] suggest potential ergogenic effects, which when combined in a product formulation could theoretically provide additional benefits over caffeine alone. However, due to the common conclusion that caffeine is the primary cause for any observed exercise performance improvement with MIPS ingestion, less attention has been given to formulations that do not include caffeine.

The relatively new category of non-caffeinated MIPS may be particularly attractive to individuals wishing to consume the products prior to evening exercise sessions. This is likely due to a desire for increased mental focus and exercise performance while also attempting to avoid the common disruption in sleep seen with evening caffeine consumption [14]. Indeed, Buman et al. [15] noted that approximately 20% of the US population exercises within 4 hours of bedtime, suggesting that the use of caffeinated MIPS may be contraindicated for an appreciable portion of the population. This finding demonstrates the need to investigate the potential efficacy of non-caffeinated MIPS, particularly when compared to similar formulations containing caffeine.

A 2017 investigation by Tinsley et al. [16] examined the effect of a caffeinated MIPS and non-caffeinated MIPS on isokinetic force production throughout 5 sets of 6 repetitions utilizing a mechanical squat device (Exerbotics eSq) in moderately resistance-trained males and females (≥2 h/week of resistance training over the previous 6 months). Collectively, the researchers determined that neither supplement condition increased squat performance in comparison to placebo. However, when examining only the male cohort, small to moderate effect sizes suggested a potential benefit of both caffeinated and non-caffeinated MIPS ingestion on concentric force production. Nonetheless, it must be noted that the MIPS utilized in the investigation varied in their formulations beyond merely caffeine content, making direct comparisons difficult. Nonetheless, to the authors’ knowledge, it remains the sole investigation directly comparing the effects of commercially available caffeinated and non-caffeinated MIPS formulations on exercise performance. Therefore, the purpose of the present study was to investigate the effects of caffeinated and non-caffeinated versions of the same commercially available MIPS product on resistance exercise performance; isometric and isokinetic squat performance; and perceptions of energy, fatigue, and focus throughout a resistance exercise protocol.

## 2. Methods

### Overview

**Figure 1.**
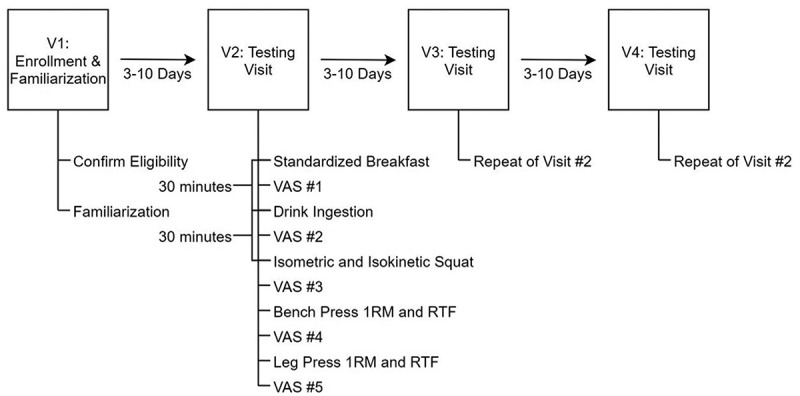
**Overview of study timeline**. *Abbreviations*: 1RM – one-repetition maximum, RTF – repetitions to failure, V – visit, VAS – visual analog scale.

The current study was a counterbalanced, placebo-controlled, double-blind investigation. Each participant completed four laboratory visits (one familiarization and screening visit and three exercise trials). For the three testing visits, participants arrived at the laboratory after an overnight fast (≥ 8 hours) from food, caffeine, dietary supplements, and other substances. Additionally, all participants were required to abstain from vigorous physical activity – defined as any activity more strenuous than a brisk walk – for at least 48 hours. Following a standard interview to assess compliance with pretesting guidelines, participants were given a standardized breakfast containing 250 kcal, 5 g fat, 45 g carbohydrate, and 9 g protein. Thirty minutes after the completion of the standardized breakfast, participants ingested either the caffeinated (C), noncaffeinated (NC), or placebo beverage from an opaque container. Additionally, participants were asked to refrain from commenting on the beverage to the research team. A single investigator was unblinded and prepared all test beverages but was not involved in any other aspect of the data collection. In addition, no other member of the research team was aware of the condition code for any research visit, nor the randomization order for any participant. After beverage ingestion, participants rested for 30 minutes before beginning the exercise testing protocol, which included performance assessments on a mechanical squat device and traditional resistance exercises. Subjective energy, focus, and fatigue were also assessed throughout the trial. The time of each performance test relative to beverage ingestion is displayed in Table 1. An outline of the study procedures is presented in Figure 1. Each of the three exercise trials was scheduled to have a minimum of 3 and a maximum of 10 days apart from one another. This study was approved by the Texas Tech University Institutional Review Board (Protocol # IRB2020-813; date of approval: 11/4/2020) and was prospectively registered on clinicaltrials.gov (Identifier: NCT04712578; first posted 1/15/2021; study start date 2/23/21).

**Table 1.** Timing of performance testing after beverage ingestion.

Performance Test	Time (min)
Isometric and Isokinetic Squat Performance	31 ± 2
Bench Press	48 ± 3
Leg Press	76 ± 6
Completion of All Performance Testing	105 ± 10

Data are presented as mean ± SD

### Participants

Twenty-four resistance-trained (≥2 resistance training session per week for 3 months prior to enrollment) college-aged males (n = 12) and females (n = 12) volunteered to participate in this study. Participant characteristics are displayed in Table 2. To be included in the present investigation, participants were required to be between the ages of 18 and 40, have a body mass between 50 and 100 kg, and be generally healthy (defined as the absence of any disease or medical condition that could potentially affect the outcomes of the study). To avoid potential negative side effects of acute caffeine consumption in non-consumers, while also matching target consumers of caffeinated MIPS, only habitual caffeine consumers (≥250 mg/day) were eligible for inclusion. Furthermore, participants were required to be able to bench press a minimum of 1.0x (males) or 0.5x (females) body mass and leg press at least 3.0x (males) or 1.75x (females) body mass. Moreover, participants were excluded for any of the following: taking a prescription medication which could reasonably make participation unsafe for the participant or influence study outcomes, self-reported caffeine sensitivity, allergy to ingredients in the test beverage or standardized breakfast, or current use of anabolic steroids.

**Table 2.** Participant characteristics.

	Males (n = 12)	Females (n = 12)
Age (yrs)	20.8 ± 2.4	21.1 ± 1.7
Height (cm)	177.2 ± 6.3	165.6 ± 6.7
Body Mass (kg)	82.7 ± 7.5	63.0 ± 10.6
Fat-Free Mass Index (kg/m^2^)	21.7 ± 1.8	16.9 ± 0.8
DXA Body Fat (%)	18.2 ± 5.8	26.0 ± 6.7
Relative Baseline Bench Press 1RM (xBM)	1.5 ± 0.2	0.8 ± 0.2
Bench Press RTF Relative to 1RM (%)	53.1 ± 9	52.3 ± 12
Relative Baseline Leg Press 1RM (xBM)	4.2 ± 0.7	2.8 ± 0.4
Leg Press RTF Relative to 1RM (%)	61.0 ± 10	54.5 ± 8
Relative Caffeine Dosage (mg/kg)	4.3 ± 0.4	5.7 ± 0.8

1RM: one repetition maximum; BM: Body mass; DXA: dual-energy X-ray absorptiometry; RTF: repetitions to failure; Data are presented as mean ± SD

### Testing procedures

#### Anthropometrics and body composition

Body composition was assessed for descriptive purposes following the pretesting guidelines interview on the first testing visit only. Body fat percentage was determined via dual-energy X-ray absorptiometry (Lunar Prodigy; General Electric, Boston, MA, USA with enCORE software v. 16.2). The reliability of this technique in our laboratory has previously been reported [17]. Body weight and height were measured via a digital scale (Seca 769, Hamburg, Germany) and stadiometer (HM200P, Charder Medical, Taichung City, Taiwan).

#### Isokinetic and isometric squat performance

Isometric and isokinetic squat performance were evaluated using a mechanical squat device (Exerbotics eSq, Tulsa, OK) [18,19]. All participants completed a standardized 5-minute warmup consisting of bodyweight squats, lunges, and jump squats prior to testing. Upon completion of the warmup, participants were instructed to remove their shoes and place their feet on the device in their normal squat stance. Foot position was recorded to allow for replication at subsequent visits. For isometric testing, the squat device was lowered to a knee angle of 120 degrees of knee extension, as determined by a manual goniometer. Participants then completed two warmup isometric pushes: one at 50% and one at 75% of perceived maximal effort. Prior to the two maximal effort isometric pushes, participants were instructed to push against the pad ‘as hard and as fast as possible’. The participant then proceeded to push upward against the pad (i.e. attempt to complete the concentric portion of a squat movement) for ~ 3 seconds. Following isometric testing, participants began the isokinetic assessments. While keeping the same foot position as was used for isometric testing, participants performed two 3-repetition sets of squats starting at 150 degrees and ending at 90 degrees of knee extension. Each squat repetition consisted of a 4-second eccentric phase, a ~ 1-second pause at the bottom of the movement, and a 4-second concentric phase. A ~ 1-second pause was also incorporated prior to the beginning of each eccentric phase. At each visit, the first set of three repetitions was performed as a warmup and to refamiliarize the participant with the squat movement. This set was completed at 50% of perceived maximal effort but was not analyzed. The first repetition of the second set was also completed at 50% of perceived maximal effort and not included in analysis, but the final two repetitions were performed with maximal effort. Strong verbal encouragement from the blinded research team was given during maximal effort repetitions. Only the final two repetitions of the second set – which were completed with maximal effort – were used for subsequent analysis.

Force data during the isometric and isokinetic tests was sampled from a load cell at 1 kHz (MP1150WSW, Biopac Systems Inc., Santa Barbara, CA) and later processed using a custom software program (LabVIEW Version 11.0, National Instruments, Austin, TX). All analyses were conducted using a scaled and filtered force signal (low-pass filtered with a 10-Hz cutoff, zero-phase lag, fourth-order Butterworth filter). To determine isometric peak force (PF_iso_), the highest 500 ms epoch was identified and quantified. In addition, peak rate of force development (RFD_peak_), early rate of force development (RFD_50_; i.e. RFD in the first 50 ms), and late rate of force development (RFD_200_; i.e. RFD in the first 200 ms) were determined. The initiation of force production for RFD variables was determined using the automated method, with the onset specified as 1% of the maximal force produced. To quantify isokinetic peak force, the highest mean 25 ms epochs for concentric and eccentric portions of the repetition were identified. These values obtained from the second and third repetitions (i.e. maximal effort repetitions) of the second 3-repetition set were averaged for analysis.

#### Resistance exercise performance

Following completion of isometric and isokinetic testing, maximal strength (one repetition maximum; 1RM) and muscular endurance (repetitions to failure; RTF) were assessed via the bench press (BP_1RM_) and leg press (LP_1RM_) exercises. The warmups and weight selection used in the 1RM testing protocol were based on guidelines provided by the National Strength and Conditioning Association as described previously [20]. Briefly, after completing a series of progressive warmup sets, 1RM attempts were then performed with a goal of obtaining the 1RM within three to five attempts. The maximal weight lifted with proper form was recorded as the participant’s 1RM. Three minutes following the completion of 1RM testing, a single set to momentary muscular failure was completed with a load of 0.75x body mass (males) or 0.4x body mass (females) for the bench press (BP_RTF_) and 2.5x body mass (males) or 1.5x body mass (females) for the leg press (LP_RTF_). The weight used for the RTF assessment during the first testing visit was used for all subsequent visits.

#### Subjective measures

Visual analog scales (VAS) for subjective ratings of energy, fatigue, and focus were collected five times throughout each visit: 1) Prior to the ingestion of the test beverage; 2) After beverage ingestion and prior to isometric and isokinetic testing; 3) Upon completion of isometric and isokinetic squat testing and prior to bench press 1RM and RTF assessments; 4) Following bench press RTF assessment and before beginning the leg press 1RM assessment; and 5) Upon completion of the entire visit, following the leg press RTF assessment. Data were collected and measured via software application (VasQ, Maki Nakata) on an electronic tablet. All VAS were grounded with relevant descriptors on either extreme of the line but no additional markings on the line itself. All values were expressed as a score ranging from 0 to 100, with zero being the minimum score and 100 being the maximal score.

### Randomization

Due to the inclusion of three conditions (C, NC, and P [Placebo]), there were six possible sequences in which participants could complete the study (i.e. C-NC-P, C-P-NC, NC-C-P, NC-P-C, P-C-NC, and P-NC-C). Based on the sample size (n = 24; n = 12 females and n = 12 males), each sequence was used exactly four times, twice in female participants and twice in male participants. The order in which the sequences were assigned to participants was determined separately for females and males using a random sequence generator within the *random* R package [21].

### Justification of sample size

An *a priori* power analysis (date: 08/27/2020) was performed using data from a previous investigation using the mechanized squat device in our laboratory [22]. The primary outcome variables were specified as PF_iso_ and RFD_peak_ at the 120-degree knee angle. Based on effect sizes observed in the previous investigation, which compared a caffeinated beverage to a placebo beverage, it was estimated that approximately 21 participants would be needed to detect a difference in both outcome variables. However, due to the number of possible condition orders (i.e. six), the target sample size was specified as 24 so that each condition order could be completed an equivalent number of times.

### Supplementation

During each study visit, participants ingested a single serving of a commercially available, caffeine-containing pre-workout supplement (22.76 g Pulse® Pre-Workout, Tropical Punch Flavor, Legion Athletics Inc.; Lot Number 320,316-01), a single serving of a stimulant-free formulation of the same product (22.05 g Pulse® Stim-Free Pre-Workout, Tropical Punch Flavor, Legion Athletics Inc.; Lot Number 720,296-01), or a placebo condition consisting of a non-caloric drink mix (2.50 g Tropical Punch Drink Mix, H-E-B Grocery Company, LP; Lot Numbers WD220, WD246, and WD247). Supplement facts for each condition are displayed in Table 3. Both pre-workout supplements were purchased via online retail orders, and the non-caloric drink mix was purchased from a local retail location. To facilitate participant blinding and flavor matching between conditions, 1.5 g of the placebo non-caloric drink mix was added to the caffeine-containing pre-workout condition, and 1.0 g of the placebo non-caloric drink mix was added to the non-caffeine condition. The inclusion of the additional 0.5 g in the caffeinated condition was to mask the characteristic bitter taste of caffeine, which was determined to be necessary by the single unblinded investigator during pre-study taste testing. Serving sizes of all supplement and placebo conditions were measured to the nearest 0.01 g using a precision digital scale (AWS-100, American Weigh Scales Inc.), which was calibrated prior to each supplement preparation session using a 100 g calibration weight. Furthermore, because recent evidence has suggested that product settling can cause the caffeine content of an individual serving of pre-workout to vary widely [23], each supplement container was vigorously agitated fifty times prior to serving size quantification during every study visit.

**Table 3.** Supplement facts.

	C	NC	P
Calories	10	5	5
Carbohydrate (g)	6	4	1
Calcium (mg)	148	236	0
Sodium (mg)	230	110	80
Potassium (mg)	345	270	40
L-Citrulline DL-Malate 2:1 (g)	8	8	–
CarnoSyn® Beta-Alanine (g)	3.6	3.6	–
Betaine Anhydrous (g)	2.5	2.5	–
Caffeine Anhydrous (mg)	350	–	–
L-Theanine (mg)	350	–	–
AlphaSize® Alpha-Glyceryl Phosphoryl Choline (GPC) 50% (mg)	300	300	–

Each supplement was mixed with 300 mL of cold water in an opaque shaker bottle. To ensure the supplement was properly dissolved, each shaker bottle was vigorously shaken fifty times, with a spherical wire whisk added to the bottle to ensure proper mixing. After agitation, 250 g of ice was added to each solution, which was then vigorously shaken another fifty times. Participants were instructed to drink the assigned beverage within a five-minute time interval without commenting on its flavor or texture for the remaining duration of the visit. To ensure complete consumption of the supplements, after participants had finished drinking, 100 mL of cold water was added to the shaker bottle and was vigorously shaken fifty times before being given back to the participant. This small volume of liquid was then consumed in a one-minute time span. Following the supplement consumption window, participants rested 30 minutes in the laboratory prior to muscular performance testing. This time interval ensured appropriate pre-exercise timing of the supplement based on manufacturer instructions.

To assess blinding efficacy, participants were instructed to report at the end of the visit which supplement condition they believed was provided to them. This was accomplished via questionnaires which were only viewed by the unblinded investigator who prepared the supplemental conditions. Participants were allowed to repeat the same guesses between visits.

### Analysis

Data were analyzed via linear mixed effects models using the *nlme* R package [24,25]. Based on the crossover design, consideration of potential carry over and period effects was warranted [26]. While we sought to minimize these effects through study design choices – including familiarization, the washout period between each visit, and balancing of condition orders – both effects were included in the mixed effects models so that the effect of supplement condition would be adjusted for these effects. Carry over effects were included in the model through inclusion of two coded variables (X1 and X2) based on which condition preceded a given condition, as previously described [27,28]. Period effects were included in the model through a Visit variable, which indicated the first, second, or third visit for each participant. Due to repeated measures within participants, a random intercept for participant was included in the model. A Sex term was also included in the model. Therefore, the overall model for outcomes with one value per condition (i.e. resistance exercise and squat variables) was represented by:

Outcome∼Condition+Sex+Condition∗Sex+Visit+X1+X2+∼1|Participant

Visual analog scale variables, which were assessed at multiple time points within each condition, included an additional Time term and interactions between Time and other variables:

Outcome∼Condition+Sex+Time+Condition∗Sex+Condition∗Time+Sex∗Time+Condition∗Sex∗Time+Visit+X1+X2+∼1|Participant

In all models, the reference groups were placebo for Condition, female for Sex, and the first visit for Visit. In the visual analog scale models, the first assessment was the reference group for Time. A first-order autoregressive (AR1) variance-covariance matrix was used when the models and intervals could be successfully produced. In cases in which intervals could not be produced due to the Hessian matrix being non-positive-definite, the default matrix of the *nlme* R package [24] was used. The correlation form was ∼1|Participant for outcomes with a single assessment per condition and was Time|Participant/Condition for visual analog scale variables. Models were fit by maximizing the restricted log-likelihood (REML). Model coefficients (i.e. *b* and associated 95% confidence intervals [CI]) were visualized using the *sjPlot* R package [29], which also provided marginal and conditional R^2^ values [30,31].

Models fit with *nlme* are allowed to exhibit within-group errors that are correlated or that have unequal variances [24]. Linearity was assessed through visual inspection of model residuals vs. predictor value plot. Normality of model residuals was assessed through visual inspection of quantile-quantile plot, supplemented by Shapiro-Wilk tests. When necessary due to normality violations, data were transformed using the *BestNormalize* R package [32]. Raw data were used for most outcomes (BP_1RM_, BP_RTF_, LP_1RM_, LP_RTF_, PF_CON_, PF_ECC_, RFD_200_, energy, focus, and fatigue), while three were transformed (RFD_peak_, PF_iso_, RFD_50_) due to violations of normality and improvement of normality after transformation. For these variables, simple log transformations or ordered quantile transformations were performed as appropriate using the *log_x* and *orderNorm* functions of the *BestNormalize* package [32].

There were no missing data for BP_1RM_, BP_RTF_, LP_1RM_, or squat variables. There was one invalid data point for LP_RTF_ due to accidental use of the incorrect load. This data point was removed and treated as missing. An additional female participant was excluded due to sensitivity analysis results indicating that the extreme data produced by the participant caused notable problems with model assumptions. Specifically, this individual performed extremely large numbers of repetitions during RTF tests (range of 73 to 208 repetitions across the three conditions). There was a total of 12 missing values (3.3%) for VAS variables, primarily due to saving errors on the electronic tablet. For missing data, multiple imputation with 100 iterations was performed using the *mice* R package [33] in order to estimate the missing values and preserve the full sample size for each outcome variable.

Statistical significance was accepted at *p* < 0.05, although additional metrics are presented to facilitate holistic interpretation of results.

## 3. Results

### Squat

**Figure 2.**
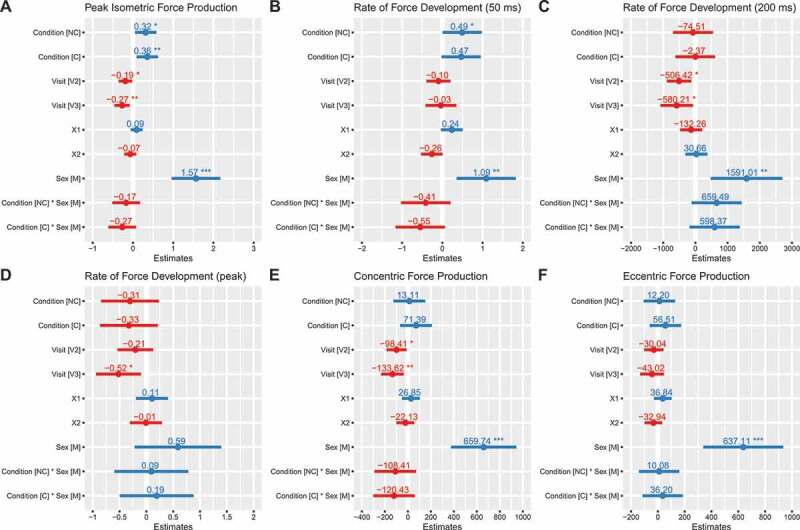
**Model coefficients for squat performance**. Linear mixed effects model coefficients (i.e. *b* and associated 95% confidence intervals) are displayed. The reference groups in the mixed model equation were female for sex, placebo for the condition, and visit 1 (V1) for the visit. Coefficients are based on simple log transformed data for isometric peak force (panel A) and RFD50 (panel B), raw data units (N/s) for RFD200 (panel C), ordered quantile transformed data for RFD_peak_ (panel D), and raw data units (N) for peak concentric and eccentric forces (panels E and F). X1 and X2 represent carryover effects. * indicates *p* ≤ 0.05; ** indicates *p* ≤ 0.01; *** indicates *p* ≤ 0.001. *Abbreviations*: C – caffeinated, NC – non-caffeinated, V2 – visit 2, V3 – visit 3, M – male.

**Figure 3.**
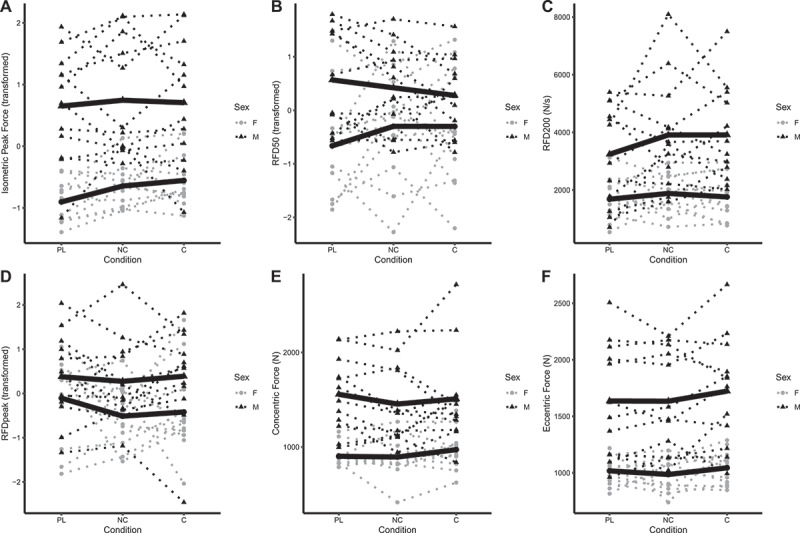
**Individual squat performance**. Each individual circle (females) and triangle (males) represents an individual participant, with dotted lines connecting each participant’s data for all three conditions. The bolded lines represent the means of each sex for each condition. Simple log transformed data are presented for isometric peak force (panel A) and RFD50 (panel B), raw data (N/s) are presented for RFD200 (panel C), ordered quantile transformed data are displayed for RFD_peak_ (panel D), and raw data (N) are displayed for peak concentric and eccentric forces (panels E and F). Note: these raw data are displayed to facilitate understanding of the dataset and individual responses but have not been adjusted for other mixed model terms. To view the adjusted effects, review the results presented in Figure 2, the main text, and the supplementary tables. *Abbreviations*: PL – placebo, NC – non-caffeinated, C – caffeinated, F – female, M – male.

PF_iso_ was higher with NC (*b*: 0.32 transformed units [95% CI: 0.05, 0.58]; *p* = 0.02) and C (*b*: 0.36 transformed units [0.09, 0.62]; *p* = 0.01) as compared to placebo (Figure 2A). Rate of force development from 0 to 50 milliseconds (RFD_50_) was higher in NC (*b*: 0.49 transformed units [95% CI: 0.01, 0.98]; *p* = 0.047) as compared to placebo, with a trend for higher values in C (*b*: 0.47 transformed units [95% CI: −0.02, 0.96]; *p* = 0.06) (Figure 2B). However, a trend was also present for the interaction between C and the male sex (*b*: −0.55 transformed units [95% CI: −1.17, 0.07]; *p* = 0.08), indicating potentially lower RFD_50_ for males in the C condition relative to the reference model. Rate of force development from 0 to 200 milliseconds (RFD_200_) did not significantly differ based on supplementation, although values were apparently greater in males during the NC (*b*: 660 N/s [−119, 1438]; *p* = 0.095 for NC×male interaction) and C (*b*: 598 N/s [−180, 1377]; *p* = 0.128 for C× male interaction) conditions (Figure 2C). RFD_peak_ did not significantly differ based on supplementation (Figure 2D). Concentric peak force (PF_CON_) did not differ significantly between placebo and C (*b*: 71 N [95% CI: −67, 210]) or NC (*b*: 13 N [−125, 152]) conditions (Figure 2E). Similarly, eccentric peak force (PF_ECC_) did not differ significantly between placebo and C (*b*: 57 N [−60, 173]) or NC (*b*: 12 N [−105, 129]) (Figure 2F). PF_iso_, RFD_50_, RFD_200_, PF_CON_ and PF_ECC_ were higher in males as compared to females (*p* ≤ 0.01). Individual responses are presented in Figure 3, and model coefficients and R^2^ values are presented in tabular form in Supplemental Tables 1 – 6. Raw (i.e. untransformed) data for PF_iso_, RFD_50_, and RFD_peak_ are displayed in Supplemental Figures 1 – 3. For squat variables, marginal R^2^ values ranged from 0.164 to 0.513.

### Resistance exercise

**Figure 4.**
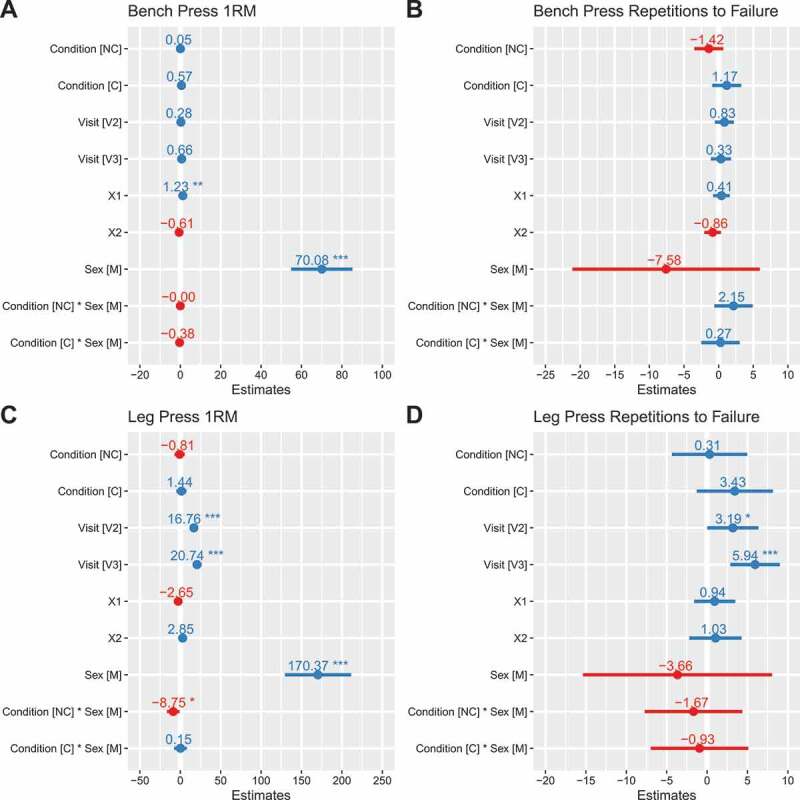
**Model coefficients for resistance exercise performance**. Linear mixed effects model coefficients (i.e. *b* and associated 95% confidence intervals) are displayed. The reference groups in the mixed model equation were female for sex, placebo for the condition, and visit 1 (V1) for the visit. Coefficients are based on raw data for one-repetition maximums (kg; panels A and C) and repetitions to failure (repetitions; panels B and D). X1 and X2 represent carryover effects. * indicates *p* ≤ 0.05; ** indicates *p* ≤ 0.01; *** indicates *p* ≤ 0.001. *Abbreviations*: C – caffeinated, NC – non-caffeinated, V2 – visit 2, V3 – visit 3, M – male.

**Figure 5.**
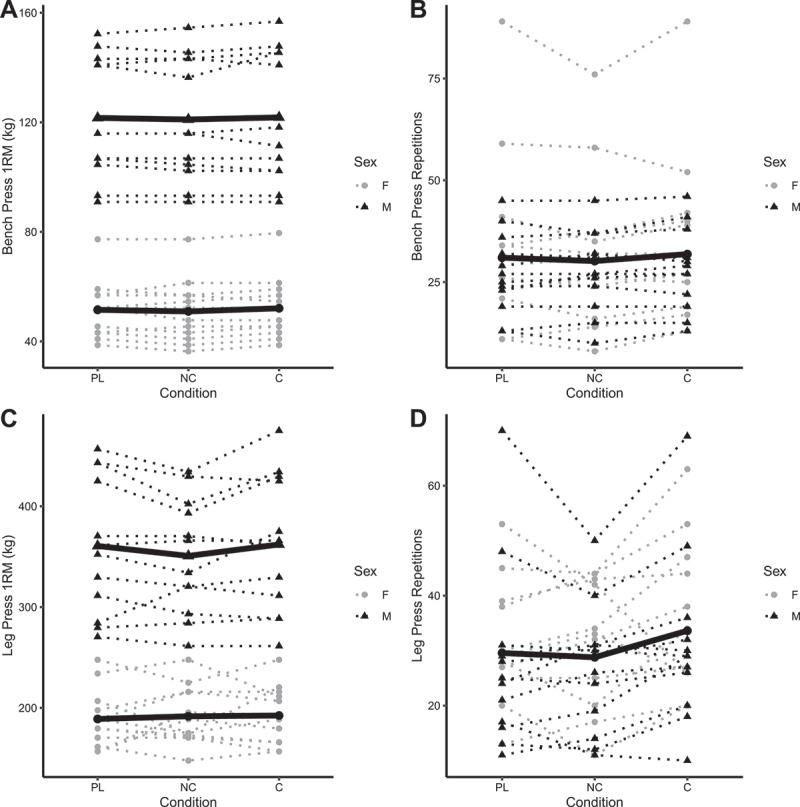
**Individual resistance exercise performance**. Each individual circle (females) and triangle (males) represents an individual participant, with dotted lines connecting each participant’s data for all three conditions. The bolded lines represent the means of each sex for each condition for bench press and leg press one-repetition maximums (panels A and C). A single bolded line, representative of mean values in both sexes combined, is presented for bench press repetitions to failure (panel B) and leg press repetitions to failure (panel C) due to no effect of sex in the linear mixed effects model analysis. Raw units (kg for one-repetition maximums and repetitions for repetitions to failure) are presented. Note: these raw data are displayed to facilitate understanding of the dataset and individual responses but have not been adjusted for other mixed model terms. To view the adjusted effects, review the results presented in Figure 4, the main text, and the supplementary tables. *Abbreviations*: PL – placebo, NC – non-caffeinated, C – caffeinated, F – female, M – male.

BP_1RM_ did not differ significantly between placebo and C (b: 0.6 kg [95% CI: −0.9, 2.1]) or NC (b: 0.1 kg [−1.5, 1.6]) conditions (Figure 4A). Similarly, BP_RTF_ did not differ significantly between placebo and C (b: 1.2 repetitions [−0.9, 3.3]) or NC (b: −1.4 repetitions [−3.5, 0.7]) conditions (Figure 4B). LP_1RM_ did not differ between placebo and C (b: 1.4 kg [−4.9, 7.7]), but was lower in the NC condition in males only (b: −8.8 kg [−16.6, −0.9]; *p* = 0.03 for NC×male interaction) (Figure 4C). LP_RTF_ did not differ from placebo for C (b: 3.4 repetitions [−1.3, 8.2]) or NC (b: 0.3 repetitions [−4.4, 5.0]) (Figure 4D). 1RM values were higher in males versus females (*p* < 0.001), but no sex differences were observed for RTF variables (*p* ≥ 0.26). Individual responses are presented in Figure 5, and model coefficients and R^2^ values are presented in tabular form in Supplemental Tables 7 – 10. For resistance exercise variables, marginal R^2^ values ranged from 0.151 to 0.998.

### Subjective measures

**Figure 6.**
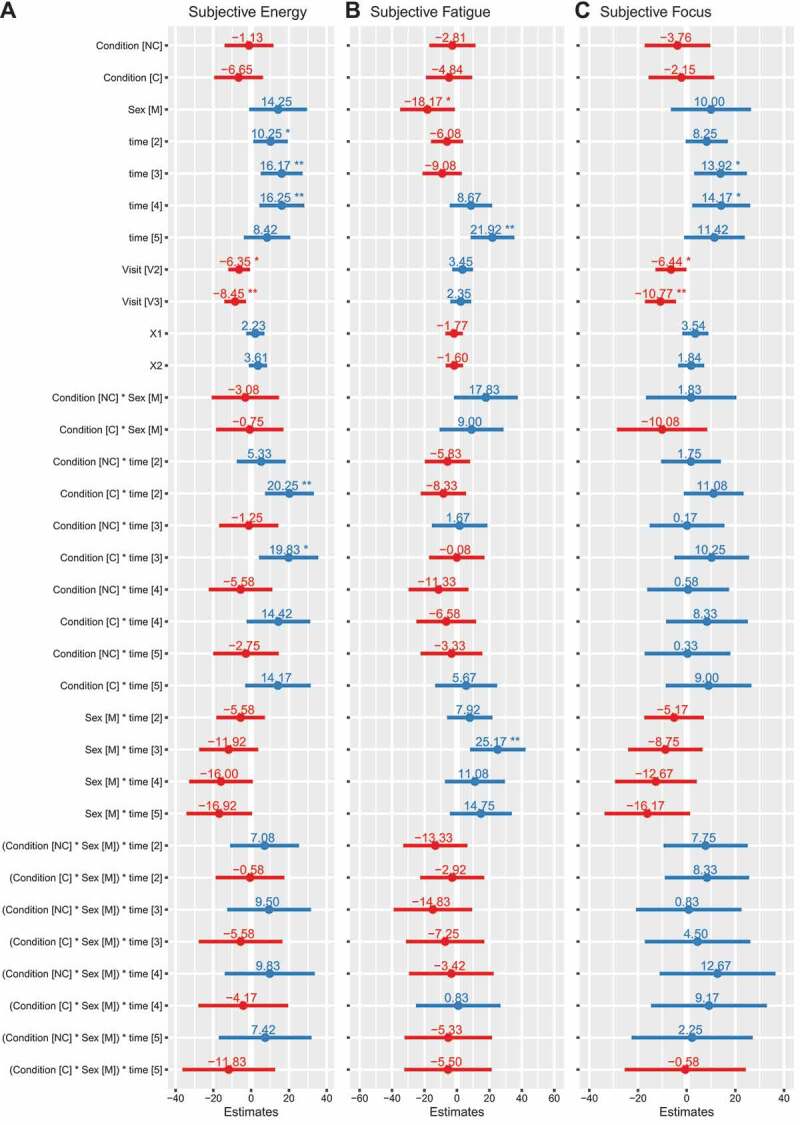
**Model coefficients for visual analog scales**. Linear mixed effects model coefficients (i.e. *b* and associated 95% confidence intervals) are displayed. The reference groups in the mixed model equation were female for sex, placebo for the condition, visit 1 (V1) for the visit, and the first/baseline assessment within a condition for time. Coefficients are based on raw data (0 to 100 mm on visual analog scale). X1 and X2 represent carryover effects. * indicates *p* ≤ 0.05; ** indicates *p* ≤ 0.01; *** indicates *p* ≤ 0.001. *Abbreviations*: C – caffeinated, NC – non-caffeinated, V2 – visit 2, V3 – visit 3, M – male.

**Figure 7.**
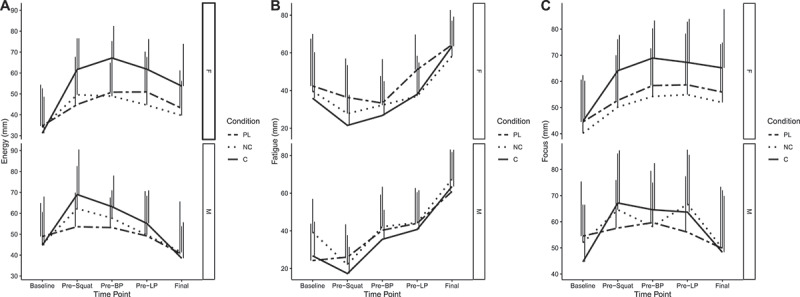
**Visual analog scale variables**. Raw values for energy (A), fatigue (B), and focus (C), as quantified by visual analog scales. The upper portion of each panel represents values in females (F), and the lower portion of each panel represents values in males (M). Major lines represent mean values across the testing protocol, and vertical lines indicate SD at each time point. Note: these raw data are displayed to facilitate understanding of the dataset and individual responses but have not been adjusted for other mixed model terms. To view the adjusted effects, review the results presented in Figure 6, the main text, and the supplementary tables. *Abbreviations*: PL – placebo, NC – non-caffeinated, C – caffeinated, F – female, M – male, BP – bench press, LP – leg press.

For energy, condition×time (*p* = 0.007) and sex×time (*p* = 0.008) interactions were present (Figure 6A). After supplement ingestion, energy ratings were higher in C (range of *b*: 14 [95% CI: −3, 32] to 20 [7, 33], *p* = 0.002 to 0.1), but not NC (range of *b: −6* [−22, 11] to 5 [−8, 18], *p* = 0.4 to 0.9), as compared to placebo, for the four post-ingestion assessments spread throughout the exercise testing battery. There were trends for greater decreases in energy for males at the final two time points in the testing battery (*b*: −16 [−33,1] and −17 [−34,0], *p* = 0.06), as compared to females. For fatigue, a sex×time interaction was present (*p* = 0.002) (Figure 6B). In males, fatigue was greater at the pre–bench press assessment (*b*: 25 [8, 42], *p* = 0.004), as compared to females. For focus, condition×time (*p* = 0.04) and sex×time (*p* = 0.005) interactions were present (Figure 6C). There was a trend for increased focus in C (*b*: 11 [−1, 23], *p* = 0.08), but not NC (*b*: 2 [−10, 14], *p* = 0.78), as compared to placebo, at the first assessment after supplement ingestion. There was also a trend for decreased focus in males at the end of the testing battery (−16 [−34, 1], *p* = 0.07), as compared to females. Changes in VAS variables over time are presented in Figure 7, and model coefficients and R^2^ values are presented in tabular form in Supplemental Tables 11 – 13. For subjective variables, marginal R^2^ values ranged from 0.247 to 0.394.

### Blinding efficacy

Fourteen out of 24 participants (58.3%) were able to correctly identify that they had consumed the caffeine condition, which was also matched by the correct identification rate of the placebo condition [14 out of 24 participants (58.3%)]. However, only 9 out of 24 participants (37.5%) correctly identified the NC condition. These rates are similar to the blinding efficacy reported by previous double-blind, placebo-controlled investigations which examined caffeine-containing products [34,35].

## 4. Discussion

The present investigation examined the influence of caffeinated and non-caffeinated versions of a commercially available MIPS formulation on squat, bench press, and leg press performance, as compared to a placebo control. Potential influences of the products on self-reported measures of energy, focus, and fatigue were also examined. The primary findings of the current study were that: 1) acute ingestion of either a caffeinated or non-caffeinated pre-workout supplement formulation improved maximal force production (PF_iso_) during an isometric squat test and may have improved early RFD (RFD_50_), particularly in females, but did not appreciably affect late RFD characteristics (RFD_200_) or RFD_peak_; 2) within the context of the laboratory environment, neither supplement provided additional benefit to leg press, bench press, or isokinetic squat performance over placebo; and 3) the consumption of the caffeinated, but not non-caffeinated, MIPS formulation increased subjective ratings of energy compared to placebo.

Interestingly, the present study did not detect differential effects of caffeine on performance outcomes, as peak isometric force production and measures of muscular endurance were similar between both MIPS formulations. However, these results somewhat mirror the findings of several previous studies comparing caffeine-containing and caffeine-free conditions [16,36]. Lane and Byrd [36] compared the acute ergogenic benefits between a commercially available MIPS formulation containing 300 mg caffeine, a caffeine-matched placebo, and an inert placebo. Though the researchers found similar but modest improvements in bench press peak velocity between the MIPS product and the caffeine-matched placebo, neither product was found to improve lower-body power production when compared to the inert placebo. Similarly, Tinsley and colleagues [16] assessed the acute ergogenic benefits of two similar MIPS formulations, though only one contained caffeine. The researchers noted that neither MIPS product resulted in lower-body performance improvements compared to placebo, though greater performance decrements during an extended lower-body testing session were identified in the caffeine-free MIPS condition. Taken together with the results of the present investigation, these findings suggest that while caffeine has acute ergogenic potential when consumed as part of a MIPS product, it may not consistently exert a large effect in the context of many laboratory tests.

As mentioned previously, peak isometric force production was improved following consumption of both caffeinated and non-caffeinated MIPS formulations compared to placebo. Since caffeine was apparently not responsible for this effect, a consideration of other ingredients is warranted. Both formulations contain 8 g citrulline malate, 3.6 g beta-alanine, 2.5 g betaine, and 300 mg alpha-glyceryl phosphoryl choline (α-GPC). However, it is unlikely that the acute performance benefits observed in the present study were mediated by betaine or beta-alanine, as these ingredients appear to require prolonged supplementation to elicit ergogenic effects [11,37]. Similarly, though citrulline malate has been shown to improve measures of muscular endurance when consumed in an acute context [38,39], it appears less effective to promote strength-related outcomes [8,39]. However, acute supplementation with 600 mg α-GPC has previously been shown to increase peak upper-body force production in resistance-trained participants, as assessed via bench press throws [40]. Though this dosage is twice the amount used by the present study, it should be noted that Hoffman and colleagues [41] also reported increased vertical jump power in a cohort of 19 recreationally active college-aged adults following the acute consumption of 150 mg of α-GPC as part of a multi-ingredient supplement. Thus, when taken together with the current data, these findings suggest that α-GPC could potentially contribute to the improvements in peak isometric force production observed during both supplemental conditions in the present investigation.

The detectable increase in PF_iso_ in both MIPS conditions may also be partially attributed to this metric being used as one of the primary outcomes for the *a priori* power analysis to determine the overall sample size of the present investigation. Statistical power may have been lower than necessary to detect small but real between-condition differences in some of the study’s other outcomes. Nonetheless, the lack of notable improvements in maximal strength during resistance exercise testing following MIPS consumption appears to be consistent with other investigations [16,42–45]. However, the peculiar finding of a suppressed LP_1RM_ in males during the NC condition warrants further discussion. In the NC condition, LP_1RM_ in male participants was lower than either C or placebo conditions (−8.75 kg [95% CI: −16.58 to −0.93 kg]). The primary mechanism by which MIPS ingestion appears to influence an acute exercise bout may be through increased muscular endurance, thus increasing individual session volume [5]. This can largely be attributed to ingredients like caffeine and citrulline malate, which can act to delay muscular fatigue throughout a workout [14,38,46,47]. However, these benefits are typically observed when multiple sets to failure are employed, not maximal strength assessments such as 1RM. For example, Bergstrom and colleagues [43] found that healthy male subjects were able to perform 9% more total body exercise volume and 14% more lower-body exercise volume during 4 sets to failure following consumption of a caffeine- and citrulline-containing MIPS compared to placebo only. However, the researchers did not detect between-condition differences in post-exercise lower-body strength and power performance. Because these outcomes were tested following the sets to fatigue, it is possible that the participants were more fatigued during post-exercise strength testing in the MIPS condition due to the greater exercise volume completed. Similarly, the lower LP_1RM_ observed in the present study could potentially be attributed to the inability to fully recover from the increased lower body performance earlier in the exercise testing session (i.e. during the PF_iso_ test, in which NC performance was greater than placebo). However, this explanation is speculative.

The present investigation did not detect an appreciable impact of either MIPS formulation on measures of muscular endurance or isokinetic performance. Indeed, the results of previous investigations examining the effects of MIPS ingestion on muscular endurance are somewhat mixed [5]. For example, Hoffman and colleagues [41] did not detect an effect of MIPS ingestion on the maximal number of push-ups or sit-ups completed within one minute. Conversely, another study by the same group reported apparently greater total back squat repetitions (51.0 ± 5 vs 47.9 ± 8.2; *p* = 0.08) following MIPS ingestion [48]. Findings of improved exercise performance have been reported by several other research groups [43,44,49–53]. However, these data contrast with the current results, likely due to differences in testing methodology. The studies displaying improved performance typically employed multiple sets, resulting in a cumulative difference over the course of the session even when significant between-condition differences were not seen for individual sets. For instance, Hoffman and colleagues [48] only reported statistically significant differences in single-set volume on set five of six, although trends for increases in total repetitions (51.0 ± 5 vs 47.9 ± 8.2; *p* = 0.08) and volume (15,939 ± 1524 kg vs 14,927 ± 2415 kg; *p* = 0.09) were also reported. Therefore, in contrast to the single set to failure employed in the present investigation, multiple sets may be needed for the effects of MIPS on muscular endurance to become apparent. As multiple set training protocols are commonplace among active individuals and athletes, quantifying the effects of MIPS over multiple sets may also enhance generalizability. Similarly, the present investigation did not identify an effect of either MIPS formulation on measures of isokinetic performance. However, these findings are in accordance with two previous investigations by our group which utilized similar supplements and the same isokinetic testing device [16,22].

The most commonly reported reason for MIPS consumption among regular users is the desire for increased energy and mental focus [6]. These data suggest that subjective energy ratings were generally higher after drink consumption for C (*p* = 0.002-0.1) but not NC (*p* = 0.4-0.9) when compared to placebo. Interestingly, C primarily appeared to increase energy at the post-ingestion (*p* = 0.002) and pre-bench press (*p* = 0.013) timepoints, the latter of which occurred roughly 48 ± 3 min minutes following beverage consumption. The general finding that caffeine increased subjective measures of energy is not surprising as this is well represented throughout the literature [14]. However, this result contrasts with a previous study by our group [16], in which consumption of a caffeinated MIPS did not result in differential effects on subjective measures of energy as part of an exercise testing battery. These divergent findings could be explained by several factors. First, despite similar caffeine content (350 mg vs. 300 mg), there were several differences in the specific formulation of the caffeinated MIPS used in the present study, such as the presence of L-theanine. The combination of caffeine and L-theanine has been shown to increase cognitive performance at dosages much lower than those found in the C condition [54–58]. Given that L-theanine was absent in the two MIPS formulations used in our prior study [16], as well as the NC condition of the present study, it is possible that L-theanine could have exerted a unique impact on this outcome. Second, methodological differences beyond the supplement formulation alone were present, which could have influenced subjective energy assessments throughout the testing battery. Lastly, it is worth noting that the general direction of change in subjective energy supported an increase in both studies, but the magnitude was smaller in the prior study – with a maximal increase of ~20 on the VAS at the post-ingestion time point – as compared to the present investigation (an increase of ≥~30 at the post-ingestion time point, as well as a subsequent time point).

The present study is not without limitations. First, all assessments were conducted in a laboratory setting. As a result, there may be limited translation to many real-world settings, in which an individual will know whether they have ingested a MIPS prior to exercise. Furthermore, while attempts were made to standardize lifestyle practices between visits, interruption of participants’ normal pre-exercise habits and environment may have impacted performance. For example, if participants habitually exercised at night after a full day of typical eating behavior, performing maximal exercise in the morning following a minimal breakfast may have prevented such participants from reaching their typical level of performance. Additionally, all participants were required to be habitual caffeine consumers. Therefore, conducting two of the three sessions in the morning after abstaining from caffeine may have influenced exercise performance. Furthermore, as the current study included only habitual caffeine consumers, our findings may not be applicable to non-consumers or those who only occasionally consume caffeine. The present investigation also did not account for sleep quality, which may have impacted the current findings. Additionally, the MIPS was provided at an absolute dosage to maximize ecological validity as opposed to a relative dosage as is commonly used in the caffeine literature. While this design choice promoted ecological validity by mimicking how MIPS are likely to be used in real-world settings, the different relative doses could have influenced our findings regarding the impact of caffeine on performance. However, average dosages in both males and females fell within the previously recommended range for caffeine efficacy (3-6 mg/kg) [14] as shown in Table 2. Furthermore, using a relative dosage of the MIPS products in the current investigation would have impacted all other ingredients. Some of these ingredients, such as citrulline and alpha-GPC, are more commonly administered in absolute rather than relative dosages. Commercially available products were used in the present study, which led to minor differences in micronutrient content between conditions. However, we do not believe these minor differences influenced the efficacy of the beverages. The current study was conducted in an acute setting; as such, these results do not provide insight into the longitudinal effects of consuming these drinks throughout a training program. Lastly, as select performance variables were used for the determination of the sample size (PF_iso,_ RFD_peak_) – one of which demonstrated a between-condition difference in the present investigation – the study may have been underpowered when examining other resistance exercise variables (e.g. BP_1RM_, BP_RTF_, LP_1RM_, LP_RTF_). Due to the growing popularity of caffeinated and non-caffeinated MIPS, future investigations should explore the potential effects of these products in longitudinal settings in conjunction with exercise protocols that closely mimic a trainee’s workout settings.

## 5. Conclusion

In conclusion, the ingestion of caffeinated or non-caffeinated MIPS increased squat isometric peak force and may have improved early RFD, particularly in females, but did not appear to appreciably augment other RFD variables, isokinetic squat, bench press, or leg press performance. Additionally, only the caffeine-containing MIPS increased perceived ratings of energy when these exercises were performed as part of a testing battery in a controlled laboratory setting. While these results indicate only select benefits of caffeinated and non-caffeinated MIPS in the laboratory context, it is acknowledged that the real-world effects of MIPS may differ due to divergent performance environments and psychological influences, such as individual knowledge of whether a MIPS has been consumed. Future longitudinal research may clarify the long-term effects of consuming caffeinated and non-caffeinated MIPS in conjunction with structured resistance training.

## Supplementary Material

Supplemental Material

Supplemental Material

Supplemental Material

Supplemental Material

## Data Availability

Data may be available from corresponding author upon reasonable request, pending relevant approvals.

## List of abbreviations

1RM – one-repetition maximum

BP – bench press

C – caffeinated MIPS

LP – leg press

MIPS – multi-ingredient pre-workout supplements

NC – non-caffeinated MIPS

NHANES – National Health and Nutrition Examination Survey

PFCON – peak concentric force production on squat device

PFECC – peak eccentric force production on squat device

PFiso – peak isometric force production on squat device

PL – placebo

RFD – rate of force development

RFD200 – rate of force development over 200 ms

RFD50 – rate of force development over 50 ms

RFDpeak – peak rate of force development

RTF – repetitions to failure

VAS – visual analog scale

α-GPC - alpha-glyceryl phosphoryl choline

## Declarations

*Ethics approval and consent to participate*

This study was approved by the Texas Tech University Institutional Review Board (Protocol # IRB2020-813; date of approval: 11/04/2020).
