# Supplementary material for: The influence of caffeinated and non-caffeinated multi-ingredient pre-workout supplements on resistance exercise performance and subjective outcomes
Source: J Int Soc Sports Nutr. 2022 Apr 4;19(1):126–49. doi: 10.1080/15502783.2022.2060048 (PMC9116396; doi:10.1080/15502783.2022.2060048)
Supplement: Supplemental Material [file RSSN_A_2060048_SM2533.docx]

**Supplemental Tables (Stratton et al.)**

Supplemental Table 1. Model coefficients for peak isometric force production.

|  | **Peak Isometric Force Production** | | |
| --- | --- | --- | --- |
| *Predictors* | *Estimates* | *CI* | *p* |
| (Intercept) | -0.78 | -1.21 – -0.35 | **0.001** |
| Condition [NC] | 0.32 | 0.05 – 0.58 | **0.021** |
| Condition [C] | 0.36 | 0.09 – 0.62 | **0.010** |
| Visit [V2] | -0.19 | -0.36 – -0.02 | **0.028** |
| Visit [V3] | -0.27 | -0.46 – -0.08 | **0.007** |
| X1 | 0.09 | -0.06 – 0.24 | 0.231 |
| X2 | -0.07 | -0.22 – 0.08 | 0.371 |
| Sex [M] | 1.57 | 0.96 – 2.17 | **<0.001** |
| Condition [NC] * Sex [M] | -0.17 | -0.51 – 0.18 | 0.330 |
| Condition [C] * Sex [M] | -0.27 | -0.61 – 0.08 | 0.127 |
| N _ID_ | 24 | | |
| Observations | 72 | | |
| Marginal R^2^ / Conditional R^2^ | 0.513 / 0.888 | | |

*Notes:* The reference groups in the mixed model equation were female for sex, placebo for the condition, and visit 1 (V1) for the visit. The summary table above was produced using the *sjPlot* R package.

*Abbreviations*: CI - 95% confidence interval, NC - non-caffeinated pre-workout supplement, C - caffeinated pre-workout supplement, V2 - visit 2, V3 - visit 3, X1 and X2 represent carryover effects, M - male, N_ID_ - number of participants.

Supplemental Table 2. Model coefficients for rate of force development over 50 ms.

|  | **Rate of Force Development (50 ms)** | | |
| --- | --- | --- | --- |
| *Predictors* | *Estimates* | *CI* | *p* |
| (Intercept) | -0.66 | -1.22 – -0.10 | **0.021** |
| Condition [NC] | 0.49 | 0.01 – 0.98 | **0.047** |
| Condition [C] | 0.47 | -0.02 – 0.96 | 0.059 |
| Visit [V2] | -0.10 | -0.40 – 0.20 | 0.523 |
| Visit [V3] | -0.03 | -0.42 – 0.35 | 0.856 |
| X1 | 0.24 | -0.03 – 0.50 | 0.085 |
| X2 | -0.26 | -0.53 – 0.01 | 0.056 |
| Sex [M] | 1.09 | 0.36 – 1.83 | **0.006** |
| Condition [NC] * Sex [M] | -0.41 | -1.03 – 0.21 | 0.185 |
| Condition [C] * Sex [M] | -0.55 | -1.17 – 0.07 | 0.080 |
| N _ID_ | 24 | | |
| Observations | 72 | | |
| Marginal R^2^ / Conditional R^2^ | 0.195 / 0.232 | | |

*Notes:* The reference groups in the mixed model equation were female for sex, placebo for the condition, and visit 1 (V1) for the visit. The summary table above was produced using the *sjPlot* R package.

*Abbreviations*: CI - 95% confidence interval, NC - non-caffeinated pre-workout supplement, C - caffeinated pre-workout supplement, V2 - visit 2, V3 - visit 3, X1 and X2 represent carryover effects, M - male, N_ID_ - number of participants.

Supplemental Table 3. Model coefficients for rate of force development over 200 ms.

|  | **Rate of Force Development (200 ms)** | | |
| --- | --- | --- | --- |
| *Predictors* | *Estimates* | *CI* | *p* |
| (Intercept) | 2113.57 | 1287.64 – 2939.49 | **<0.001** |
| Condition [NC] | -74.51 | -694.13 – 545.12 | 0.809 |
| Condition [C] | -2.37 | -621.99 – 617.26 | 0.994 |
| Visit [V2] | -506.42 | -889.13 – -123.72 | **0.011** |
| Visit [V3] | -580.21 | -1088.45 – -71.97 | **0.026** |
| X1 | -132.26 | -476.11 – 211.60 | 0.442 |
| X2 | 30.66 | -313.20 – 374.52 | 0.858 |
| Sex [M] | 1591.01 | 476.57 – 2705.45 | **0.007** |
| Condition [NC] * Sex [M] | 659.49 | -119.32 – 1438.30 | 0.095 |
| Condition [C] * Sex [M] | 598.37 | -180.44 – 1377.17 | 0.128 |
| N _ID_ | 24 | | |
| Observations | 72 | | |
| Marginal R^2^ / Conditional R^2^ | 0.385 / 0.385 | | |

*Notes:* The reference groups in the mixed model equation were female for sex, placebo for the condition, and visit 1 (V1) for the visit. The summary table above was produced using the *sjPlot* R package.

*Abbreviations*: CI - 95% confidence interval, NC - non-caffeinated pre-workout supplement, C - caffeinated pre-workout supplement, V2 - visit 2, V3 - visit 3, X1 and X2 represent carryover effects, M - male, N_ID_ - number of participants.

Supplemental Table 4. Model coefficients for peak rate of force development.

|  | **Rate of Force Development (peak)** | | |
| --- | --- | --- | --- |
| *Predictors* | *Estimates* | *CI* | *p* |
| (Intercept) | 0.11 | -0.50 – 0.72 | 0.717 |
| Condition [NC] | -0.31 | -0.85 – 0.23 | 0.257 |
| Condition [C] | -0.33 | -0.87 – 0.21 | 0.230 |
| Visit [V2] | -0.21 | -0.54 – 0.13 | 0.221 |
| Visit [V3] | -0.52 | -0.94 – -0.10 | **0.017** |
| X1 | 0.11 | -0.19 – 0.40 | 0.480 |
| X2 | -0.01 | -0.31 – 0.29 | 0.961 |
| Sex [M] | 0.59 | -0.22 – 1.40 | 0.144 |
| Condition [NC] * Sex [M] | 0.09 | -0.59 – 0.78 | 0.786 |
| Condition [C] * Sex [M] | 0.19 | -0.50 – 0.88 | 0.575 |
| N _ID_ | 24 | | |
| Observations | 72 | | |
| Marginal R^2^ / Conditional R^2^ | 0.164 / 0.308 | | |

*Notes:* The reference groups in the mixed model equation were female for sex, placebo for the condition, and visit 1 (V1) for the visit. The summary table above was produced using the *sjPlot* R package.

*Abbreviations*: CI - 95% confidence interval, NC - non-caffeinated pre-workout supplement, C - caffeinated pre-workout supplement, V2 - visit 2, V3 - visit 3, X1 and X2 represent carryover effects, M - male, N_ID_ - number of participants.

Supplemental Table 5. Model coefficients for concentric force production.

|  | **Concentric Force Production** | | |
| --- | --- | --- | --- |
| *Predictors* | *Estimates* | *CI* | *p* |
| (Intercept) | 971.87 | 766.86 – 1176.88 | **<0.001** |
| Condition [NC] | 13.11 | -125.40 – 151.61 | 0.849 |
| Condition [C] | 71.39 | -67.11 – 209.90 | 0.304 |
| Visit [V2] | -98.41 | -186.28 – -10.54 | **0.029** |
| Visit [V3] | -133.62 | -233.06 – -34.19 | **0.010** |
| X1 | 26.85 | -51.18 – 104.87 | 0.491 |
| X2 | -22.13 | -100.16 – 55.90 | 0.570 |
| Sex [M] | 659.74 | 375.91 – 943.57 | **<0.001** |
| Condition [NC] * Sex [M] | -108.41 | -289.40 – 72.57 | 0.233 |
| Condition [C] * Sex [M] | -120.43 | -301.42 – 60.55 | 0.186 |
| N _ID_ | 24 | | |
| Observations | 72 | | |
| Marginal R^2^ / Conditional R^2^ | 0.447 / 0.846 | | |

*Notes:* The reference groups in the mixed model equation were female for sex, placebo for the condition, and visit 1 (V1) for the visit. The summary table above was produced using the *sjPlot* R package.

*Abbreviations*: CI - 95% confidence interval, NC - non-caffeinated pre-workout supplement, C - caffeinated pre-workout supplement, V2 - visit 2, V3 - visit 3, X1 and X2 represent carryover effects, M - male, N_ID_ - number of participants.

Supplemental Table 6. Model coefficients for eccentric force production.

|  | **Eccentric Force Production** | | |
| --- | --- | --- | --- |
| *Predictors* | *Estimates* | *CI* | *p* |
| (Intercept) | 1015.21 | 802.74 – 1227.69 | **<0.001** |
| Condition [NC] | 12.20 | -104.66 – 129.06 | 0.834 |
| Condition [C] | 56.51 | -60.35 – 173.37 | 0.334 |
| Visit [V2] | -30.04 | -102.68 – 42.60 | 0.408 |
| Visit [V3] | -43.02 | -131.70 – 45.66 | 0.333 |
| X1 | 36.84 | -28.66 – 102.33 | 0.262 |
| X2 | -32.94 | -98.43 – 32.55 | 0.315 |
| Sex [M] | 637.11 | 338.82 – 935.41 | **<0.001** |
| Condition [NC] * Sex [M] | 10.08 | -140.17 – 160.33 | 0.893 |
| Condition [C] * Sex [M] | 36.20 | -114.06 – 186.45 | 0.629 |
| N _ID_ | 24 | | |
| Observations | 72 | | |
| Marginal R^2^ / Conditional R^2^ | 0.467 / 0.871 | | |

*Notes:* The reference groups in the mixed model equation were female for sex, placebo for the condition, and visit 1 (V1) for the visit. The summary table above was produced using the *sjPlot* R package.

*Abbreviations*: CI - 95% confidence interval, NC - non-caffeinated pre-workout supplement, C - caffeinated pre-workout supplement, V2 - visit 2, V3 - visit 3, X1 and X2 represent carryover effects, M - male, N_ID_ - number of participants.

Supplemental Table 7. Model coefficients for bench press 1-repetition maximum.

|  | **Bench Press 1RM** | | |
| --- | --- | --- | --- |
| *Predictors* | *Estimates* | *CI* | *p* |
| (Intercept) | 50.99 | 40.52 – 61.46 | **<0.001** |
| Condition [NC] | 0.05 | -1.45 – 1.55 | 0.945 |
| Condition [C] | 0.57 | -0.93 – 2.07 | 0.450 |
| Visit [V2] | 0.28 | -0.71 – 1.28 | 0.568 |
| Visit [V3] | 0.66 | -0.34 – 1.67 | 0.189 |
| X1 | 1.23 | 0.37 – 2.10 | **0.006** |
| X2 | -0.61 | -1.47 – 0.26 | 0.163 |
| Sex [M] | 70.08 | 54.91 – 85.24 | **<0.001** |
| Condition [NC] * Sex [M] | -0.00 | -2.00 – 2.00 | 0.998 |
| Condition [C] * Sex [M] | -0.38 | -2.38 – 1.62 | 0.704 |
| N _ID_ | 24 | | |
| Observations | 72 | | |
| Marginal R^2^ / Conditional R^2^ | 0.998 / NA | | |

*Notes:* The reference groups in the mixed model equation were female for sex, placebo for the condition, and visit 1 (V1) for the visit. The summary table above was produced using the *sjPlot* R package.

*Abbreviations*: 1RM - one-repetition maximum, CI - 95% confidence interval, NC - non-caffeinated pre-workout supplement, C - caffeinated pre-workout supplement, V2 - visit 2, V3 - visit 3, X1 and X2 represent carryover effects, M - male, N_ID_ - number of participants.

Supplemental Table 8. Model coefficients for bench press repetitions to failure.

|  | **Bench Press Repetitions to Failure** | | |
| --- | --- | --- | --- |
| *Predictors* | *Estimates* | *CI* | *p* |
| (Intercept) | 34.10 | 24.73 – 43.47 | **<0.001** |
| Condition [NC] | -1.42 | -3.51 – 0.68 | 0.179 |
| Condition [C] | 1.17 | -0.93 – 3.26 | 0.267 |
| Visit [V2] | 0.83 | -0.53 – 2.19 | 0.223 |
| Visit [V3] | 0.33 | -1.11 – 1.78 | 0.643 |
| X1 | 0.41 | -0.79 – 1.61 | 0.495 |
| X2 | -0.86 | -2.06 – 0.34 | 0.155 |
| Sex [M] | -7.58 | -21.12 – 5.95 | 0.258 |
| Condition [NC] * Sex [M] | 2.15 | -0.62 – 4.92 | 0.125 |
| Condition [C] * Sex [M] | 0.27 | -2.50 – 3.04 | 0.845 |
| N _ID_ | 24 | | |
| Observations | 72 | | |
| Marginal R^2^ / Conditional R^2^ | 0.670 / NA | | |

*Notes:* The reference groups in the mixed model equation were female for sex, placebo for the condition, and visit 1 (V1) for the visit. The summary table above was produced using the *sjPlot* R package.

*Abbreviations*: CI - 95% confidence interval, NC - non-caffeinated pre-workout supplement, C - caffeinated pre-workout supplement, V2 - visit 2, V3 - visit 3, X1 and X2 represent carryover effects, M - male, N_ID_ - number of participants.

Supplemental Table 9. Model coefficients for leg press 1-repetition maximum.

|  | **Leg Press 1RM** | | |
| --- | --- | --- | --- |
| *Predictors* | *Estimates* | *CI* | *p* |
| (Intercept) | 177.97 | 149.61 – 206.34 | **<0.001** |
| Condition [NC] | -0.81 | -7.11 – 5.49 | 0.795 |
| Condition [C] | 1.44 | -4.86 – 7.74 | 0.646 |
| Visit [V2] | 16.76 | 12.86 – 20.67 | **<0.001** |
| Visit [V3] | 20.74 | 15.24 – 26.23 | **<0.001** |
| X1 | -2.65 | -6.14 – 0.85 | 0.134 |
| X2 | 2.85 | -0.64 – 6.35 | 0.107 |
| Sex [M] | 170.37 | 129.48 – 211.25 | **<0.001** |
| Condition [NC] * Sex [M] | -8.75 | -16.58 – -0.93 | **0.029** |
| Condition [C] * Sex [M] | 0.15 | -7.67 – 7.97 | 0.969 |
| N _ID_ | 24 | | |
| Observations | 72 | | |
| Marginal R^2^ / Conditional R^2^ | 0.754 / 0.754 | | |

*Notes:* The reference groups in the mixed model equation were female for sex, placebo for the condition, and visit 1 (V1) for the visit. The summary table above was produced using the *sjPlot* R package.

*Abbreviations*: 1RM - one-repetition maximum, CI - 95% confidence interval, NC - non-caffeinated pre-workout supplement, C - caffeinated pre-workout supplement, V2 - visit 2, V3 - visit 3, X1 and X2 represent carryover effects, M - male, N_ID_ - number of participants.Supplemental Table 10. Model coefficients for leg press repetitions to failure.

|  | **Leg Press Repetitions to Failure** | | |
| --- | --- | --- | --- |
| *Predictors* | *Estimates* | *CI* | *p* |
| (Intercept) | 28.71 | 20.26 – 37.16 | **<0.001** |
| Condition [NC] | 0.31 | -4.36 – 4.97 | 0.894 |
| Condition [C] | 3.43 | -1.28 – 8.15 | 0.149 |
| Visit [V2] | 3.19 | 0.02 – 6.36 | **0.048** |
| Visit [V3] | 5.94 | 2.88 – 9.00 | **<0.001** |
| X1 | 0.94 | -1.61 – 3.49 | 0.460 |
| X2 | 1.03 | -2.20 – 4.27 | 0.521 |
| Sex [M] | -3.66 | -15.37 – 8.05 | 0.523 |
| Condition [NC] * Sex [M] | -1.67 | -7.72 – 4.38 | 0.579 |
| Condition [C] * Sex [M] | -0.93 | -6.98 – 5.11 | 0.756 |
| N _ID_ | 23 | | |
| Observations | 69 | | |
| Marginal R^2^ / Conditional R^2^ | 0.151 / NA | | |

*Notes:* The reference groups in the mixed model equation were female for sex, placebo for the condition, and visit 1 (V1) for the visit. The summary table above was produced using the *sjPlot* R package.

*Abbreviations*: CI - 95% confidence interval, NC - non-caffeinated pre-workout supplement, C - caffeinated pre-workout supplement, V2 - visit 2, V3 - visit 3, X1 and X2 represent carryover effects, M - male, N_ID_ - number of participants.

Supplemental Table 11. Model coefficients for subjective energy.

|  | **Subjective Energy** | | |
| --- | --- | --- | --- |
| *Predictors* | *Estimates* | *CI* | *p* |
| (Intercept) | 40.80 | 29.92 – 51.69 | **<0.001** |
| Condition [NC] | -1.13 | -14.03 – 11.77 | 0.863 |
| Condition [C] | -6.65 | -19.55 – 6.25 | 0.311 |
| Sex [M] | 14.25 | -1.02 – 29.52 | 0.066 |
| time [2] | 10.25 | 1.14 – 19.36 | **0.028** |
| time [3] | 16.17 | 5.09 – 27.24 | **0.004** |
| time [4] | 16.25 | 4.36 – 28.14 | **0.008** |
| time [5] | 8.42 | -3.85 – 20.68 | 0.178 |
| Visit [V2] | -6.35 | -12.08 – -0.62 | **0.030** |
| Visit [V3] | -8.45 | -14.18 – -2.72 | **0.004** |
| X1 | 2.23 | -2.56 – 7.01 | 0.361 |
| X2 | 3.61 | -1.17 – 8.40 | 0.139 |
| Condition [NC] * Sex [M] | -3.08 | -20.90 – 14.73 | 0.734 |
| Condition [C] * Sex [M] | -0.75 | -18.57 – 17.07 | 0.934 |
| Condition [NC] * time [2] | 5.33 | -7.55 – 18.22 | 0.416 |
| Condition [C] * time [2] | 20.25 | 7.37 – 33.13 | **0.002** |
| Condition [NC] * time [3] | -1.25 | -16.91 – 14.41 | 0.875 |
| Condition [C] * time [3] | 19.83 | 4.18 – 35.49 | **0.013** |
| Condition [NC] * time [4] | -5.58 | -22.41 – 11.24 | 0.514 |
| Condition [C] * time [4] | 14.42 | -2.41 – 31.24 | 0.093 |
| Condition [NC] * time [5] | -2.75 | -20.10 – 14.60 | 0.755 |
| Condition [C] * time [5] | 14.17 | -3.18 – 31.52 | 0.109 |
| Sex [M] * time [2] | -5.58 | -18.47 – 7.30 | 0.394 |
| Sex [M] * time [3] | -11.92 | -27.57 – 3.74 | 0.135 |
| Sex [M] * time [4] | -16.00 | -32.82 – 0.82 | 0.062 |
| Sex [M] * time [5] | -16.92 | -34.27 – 0.43 | 0.056 |
| (Condition [NC] * Sex [M]) * time [2] | 7.08 | -11.14 – 25.30 | 0.445 |
| (Condition [C] * Sex [M]) * time [2] | -0.58 | -18.80 – 17.64 | 0.950 |
| (Condition [NC] * Sex [M]) * time [3] | 9.50 | -12.64 – 31.64 | 0.399 |
| (Condition [C] * Sex [M]) * time [3] | -5.58 | -27.73 – 16.56 | 0.620 |
| (Condition [NC] * Sex [M]) * time [4] | 9.83 | -13.96 – 33.62 | 0.417 |
| (Condition [C] * Sex [M]) * time [4] | -4.17 | -27.96 – 19.62 | 0.731 |
| (Condition [NC] * Sex [M]) * time [5] | 7.42 | -17.12 – 31.95 | 0.552 |
| (Condition [C] * Sex [M]) * time [5] | -11.83 | -36.37 – 12.70 | 0.343 |
| N _ID_ | 24 | | |
| Observations | 360 | | |
| Marginal R^2^ / Conditional R^2^ | 0.320 / NA | | |

*Notes:* The reference groups in the mixed model equation were female for sex, placebo for the condition, visit 1 (V1) for the visit, and the first/baseline assessment within a condition for time. The summary table above was produced using the *sjPlot* R package.

*Abbreviations*: CI - 95% confidence interval, NC - non-caffeinated pre-workout supplement, C - caffeinated pre-workout supplement, V2 - visit 2, V3 - visit 3, X1 and X2 represent carryover effects, M - male, N_ID_ - number of participants.

Supplemental Table 12. Model coefficients for subjective fatigue.

|  | **Subjective Fatigue** | | |
| --- | --- | --- | --- |
| *Predictors* | *Estimates* | *CI* | *p* |
| (Intercept) | 39.95 | 27.93 – 51.97 | **<0.001** |
| Condition [NC] | -2.81 | -17.05 – 11.44 | 0.699 |
| Condition [C] | -4.84 | -19.09 – 9.40 | 0.504 |
| Sex [M] | -18.17 | -35.00 – -1.33 | **0.036** |
| time [2] | -6.08 | -15.96 – 3.80 | 0.227 |
| time [3] | -9.08 | -21.16 – 3.00 | 0.140 |
| time [4] | 8.67 | -4.37 – 21.70 | 0.192 |
| time [5] | 21.92 | 8.44 – 35.40 | **0.002** |
| Visit [V2] | 3.45 | -2.96 – 9.86 | 0.291 |
| Visit [V3] | 2.35 | -4.06 – 8.76 | 0.472 |
| X1 | -1.77 | -7.12 – 3.58 | 0.516 |
| X2 | -1.60 | -6.95 – 3.75 | 0.557 |
| Condition [NC] * Sex [M] | 17.83 | -1.83 – 37.50 | 0.075 |
| Condition [C] * Sex [M] | 9.00 | -10.66 – 28.66 | 0.368 |
| Condition [NC] * time [2] | -5.83 | -19.80 – 8.14 | 0.412 |
| Condition [C] * time [2] | -8.33 | -22.30 – 5.64 | 0.241 |
| Condition [NC] * time [3] | 1.67 | -15.42 – 18.75 | 0.848 |
| Condition [C] * time [3] | -0.08 | -17.17 – 17.00 | 0.992 |
| Condition [NC] * time [4] | -11.33 | -29.76 – 7.10 | 0.227 |
| Condition [C] * time [4] | -6.58 | -25.01 – 11.85 | 0.483 |
| Condition [NC] * time [5] | -3.33 | -22.40 – 15.73 | 0.731 |
| Condition [C] * time [5] | 5.67 | -13.40 – 24.73 | 0.559 |
| Sex [M] * time [2] | 7.92 | -6.05 – 21.89 | 0.266 |
| Sex [M] * time [3] | 25.17 | 8.08 – 42.25 | **0.004** |
| Sex [M] * time [4] | 11.08 | -7.35 – 29.51 | 0.238 |
| Sex [M] * time [5] | 14.75 | -4.31 – 33.81 | 0.129 |
| (Condition [NC] * Sex [M]) * time [2] | -13.33 | -33.09 – 6.43 | 0.185 |
| (Condition [C] * Sex [M]) * time [2] | -2.92 | -22.68 – 16.84 | 0.772 |
| (Condition [NC] * Sex [M]) * time [3] | -14.83 | -38.99 – 9.33 | 0.228 |
| (Condition [C] * Sex [M]) * time [3] | -7.25 | -31.41 – 16.91 | 0.555 |
| (Condition [NC] * Sex [M]) * time [4] | -3.42 | -29.48 – 22.65 | 0.797 |
| (Condition [C] * Sex [M]) * time [4] | 0.83 | -25.23 – 26.90 | 0.950 |
| (Condition [NC] * Sex [M]) * time [5] | -5.33 | -32.29 – 21.63 | 0.697 |
| (Condition [C] * Sex [M]) * time [5] | -5.50 | -32.46 – 21.46 | 0.688 |
| N _ID_ | 24 | | |
| Observations | 360 | | |
| Marginal R^2^ / Conditional R^2^ | 0.394 / NA | | |

*Notes:* The reference groups in the mixed model equation were female for sex, placebo for the condition, visit 1 (V1) for the visit, and the first/baseline assessment within a condition for time. The summary table above was produced using the *sjPlot* R package.

*Abbreviations*: CI - 95% confidence interval, NC - non-caffeinated pre-workout supplement, C - caffeinated pre-workout supplement, V2 - visit 2, V3 - visit 3, X1 and X2 represent carryover effects, M - male, N_ID_ - number of participants.

Supplemental Table 13. Model coefficients for subjective focus.

|  | **Subjective Focus** | | |
| --- | --- | --- | --- |
| *Predictors* | *Estimates* | *CI* | *p* |
| (Intercept) | 50.85 | 39.11 – 62.59 | **<0.001** |
| Condition [NC] | -3.76 | -17.25 – 9.72 | 0.583 |
| Condition [C] | -2.15 | -15.64 – 11.33 | 0.753 |
| Sex [M] | 10.00 | -6.40 – 26.40 | 0.219 |
| time [2] | 8.25 | -0.41 – 16.91 | 0.062 |
| time [3] | 13.92 | 3.09 – 24.75 | **0.012** |
| time [4] | 14.17 | 2.28 – 26.05 | **0.020** |
| time [5] | 11.42 | -1.02 – 23.86 | 0.072 |
| Visit [V2] | -6.44 | -12.83 – -0.06 | **0.048** |
| Visit [V3] | -10.77 | -17.16 – -4.38 | **0.001** |
| X1 | 3.54 | -1.80 – 8.89 | 0.193 |
| X2 | 1.84 | -3.51 – 7.18 | 0.500 |
| Condition [NC] * Sex [M] | 1.83 | -16.73 – 20.40 | 0.846 |
| Condition [C] * Sex [M] | -10.08 | -28.65 – 8.48 | 0.286 |
| Condition [NC] * time [2] | 1.75 | -10.49 – 13.99 | 0.779 |
| Condition [C] * time [2] | 11.08 | -1.16 – 23.33 | 0.076 |
| Condition [NC] * time [3] | 0.17 | -15.15 – 15.48 | 0.983 |
| Condition [C] * time [3] | 10.25 | -5.07 – 25.57 | 0.189 |
| Condition [NC] * time [4] | 0.58 | -16.22 – 17.39 | 0.946 |
| Condition [C] * time [4] | 8.33 | -8.47 – 25.14 | 0.330 |
| Condition [NC] * time [5] | 0.33 | -17.26 – 17.92 | 0.970 |
| Condition [C] * time [5] | 9.00 | -8.59 – 26.59 | 0.315 |
| Sex [M] * time [2] | -5.17 | -17.41 – 7.08 | 0.407 |
| Sex [M] * time [3] | -8.75 | -24.07 – 6.57 | 0.262 |
| Sex [M] * time [4] | -12.67 | -29.47 – 4.14 | 0.139 |
| Sex [M] * time [5] | -16.17 | -33.76 – 1.42 | 0.072 |
| (Condition [NC] * Sex [M]) * time [2] | 7.75 | -9.57 – 25.07 | 0.379 |
| (Condition [C] * Sex [M]) * time [2] | 8.33 | -8.98 – 25.65 | 0.344 |
| (Condition [NC] * Sex [M]) * time [3] | 0.83 | -20.83 – 22.49 | 0.940 |
| (Condition [C] * Sex [M]) * time [3] | 4.50 | -17.16 – 26.16 | 0.683 |
| (Condition [NC] * Sex [M]) * time [4] | 12.67 | -11.10 – 36.43 | 0.295 |
| (Condition [C] * Sex [M]) * time [4] | 9.17 | -14.60 – 32.93 | 0.448 |
| (Condition [NC] * Sex [M]) * time [5] | 2.25 | -22.63 – 27.13 | 0.859 |
| (Condition [C] * Sex [M]) * time [5] | -0.58 | -25.46 – 24.30 | 0.963 |
| N _ID_ | 24 | | |
| Observations | 360 | | |
| Marginal R^2^ / Conditional R^2^ | 0.247 / NA | | |

*Notes:* The reference groups in the mixed model equation were female for sex, placebo for the condition, visit 1 (V1) for the visit, and the first/baseline assessment within a condition for time. The summary table above was produced using the *sjPlot* R package.

*Abbreviations*: CI - 95% confidence interval, NC - non-caffeinated pre-workout supplement, C - caffeinated pre-workout supplement, V2 - visit 2, V3 - visit 3, X1 and X2 represent carryover effects, M - male, N_ID_ - number of participants.
