# Supplementary figures and images for: The influence of caffeinated and non-caffeinated multi-ingredient pre-workout supplements on resistance exercise performance and subjective outcomes
Source: J Int Soc Sports Nutr. 2022 Apr 4;19(1):126–49. doi: 10.1080/15502783.2022.2060048 (PMC9116396; doi:10.1080/15502783.2022.2060048)

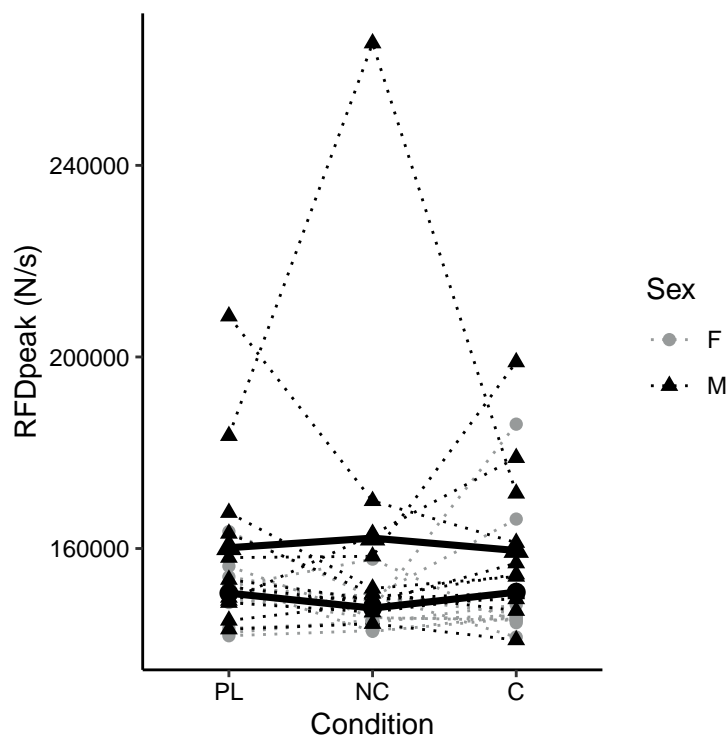

Supplement: Supplemental Material [file RSSN_A_2060048_SM2532.pdf]

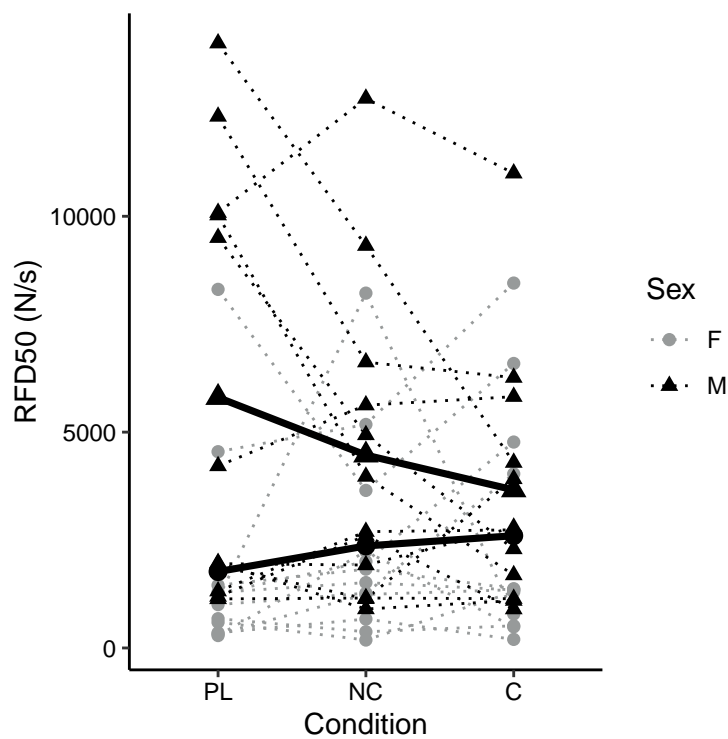

Supplement: Supplemental Material [file RSSN_A_2060048_SM2531.pdf]

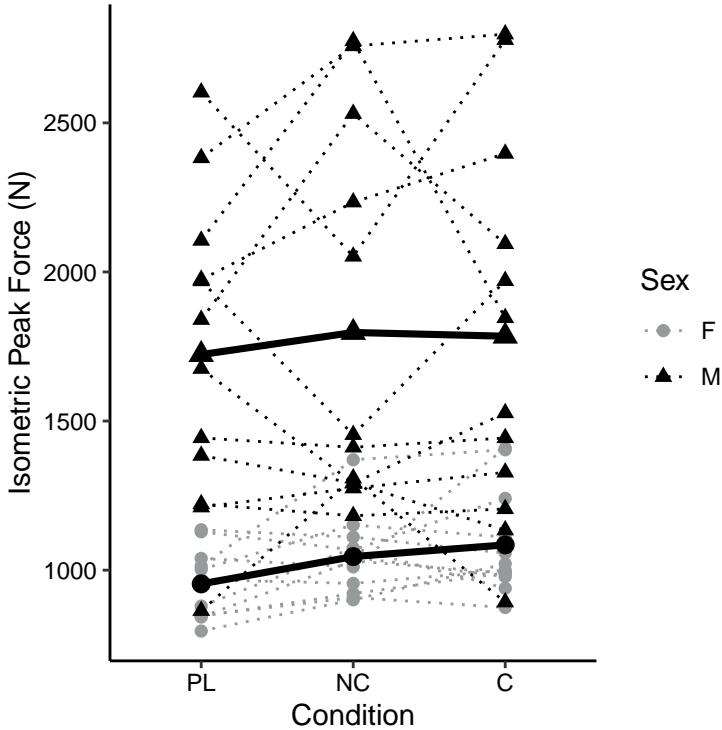

Supplement: Supplemental Material [file RSSN_A_2060048_SM2529.pdf]
